# Phylogenetic Analysis and Virulence Characteristics of Methicillin-Resistant Staphylococcus aureus ST45 in China: a Hyper-Virulent Clone Associated with Bloodstream Infections

**DOI:** 10.1128/msystems.00029-23

**Published:** 2023-03-06

**Authors:** Xinyi Wang, Xiaocui Wu, Li Shen, Lulin Rao, Bingjie Wang, Huilin Zhao, Jiao Zhang, Yanghua Xiao, Yinjuan Guo, Yanlei Xu, Liang Chen, Fangyou Yu

**Affiliations:** a Department of Clinical Laboratory, Shanghai Pulmonary Hospital, School of Medicine, Tongji University, Shanghai, China; b Center for Discovery and Innovation, Hackensack Meridian Health, Nutley, New Jersey, USA; c Department of Medical Sciences, Hackensack Meridian School of Medicine, Nutley, New Jersey, USA; Zhejiang University School of Medicine

**Keywords:** MRSA ST45, bloodstream infection, epidemic, virulence, whole-genome sequencing

## Abstract

Methicillin-resistant Staphylococcus aureus (MRSA) sequence type 45 (ST45) was rarely found in China. This study was conducted to trace the transmission and evolution of emerging MRSA ST45 strains in mainland China and explore its virulence. A total of 27 ST45 isolates were included for whole-genome sequencing and genetic characteristic analysis. Epidemiological results showed that MRSA ST45 isolates were often obtained from blood, primarily originated in Guangzhou, and carried diverse virulence and drug resistance genes. Staphylococcal cassette chromosome *mec* type IV (SCC*mec* IV) dominated in MRSA ST45 (23/27, 85.2%). ST45-SCC*mec* V was located on a phylogenetic clade distinct from the SCC*mec* IV cluster. We selected two representative isolates, MR370 (ST45-SCC*mec* IV) and MR387 (ST45-SCC*mec* V), and performed hemolysin activity, a blood killing assay, a Galleria mellonella infection model, and a mouse bacteremia model, as well as real-time fluorescence quantitative PCR. MR370 was proved to have extreme virulence in the phenotypic assays and at the mRNA level compared with ST59, ST5, and USA300 MRSA strains. MR387 was comparable to USA300-LAC on the phenotype and was verified to have higher expression of *scn*, *chp*, *sak*, *saeR*, *agrA*, and *RNAIII* than USA300-LAC. The results emphasized the extraordinary performance of MR370 and the good potential of MR387 in virulence causing bloodstream infection. Meanwhile, we conclude that China MRSA ST45 showed two different clonotypes, which may be widespread in the future. The entire study is valuable as a timely reminder and reports virulence phenotypes of China MRSA ST45 for the first time.

**IMPORTANCE** Methicillin-resistant Staphylococcus aureus ST45 is epidemic worldwide. This study contributed to the awareness of the Chinese hyper-virulent MRSA ST45 strains and served as a timely reminder of its wide dissemination of clonotypes. Further, we provide novel insights for prevention from the perspective of bloodstream infections. ST45-SCC*mec* V is a clonotype deserving special attention in China, and we performed genetic and phenotypic analyses for the first time on it.

## INTRODUCTION

Staphylococcus aureus is a common pathogen which can cause various infectious diseases, including bacteremia (1). The prevalence of methicillin-resistant S. aureus (MRSA) is a major public health concern, which remains persistently high in China (2). Sequence types 45 (ST45) is a major international representative of the epidemic MRSA (EMRSA) group (3), with huge strain diversity and a high clinical impact, which frequently caused severe invasive disease, such as bacteremia (4). ST45 is characterized by its host diversity, so transmission of potential pathogens between different hosts could be a growing public health threat (5). Therefore, the presence and spread of MRSA ST45 cannot be overlooked. The ST45 population studied contained two different sublineages separated by strong geographical signatures (4). The larger sublineage mainly consisted of isolates from Europe and North America, while the other was mainly correlated with Australia (4). Isolates originating from Asia could be found in both sublineages, but in small quantities (4). In brief, ST45 was isolated worldwide and particularly predominant in North America, Europe, and Australia (6), while less frequently reported in Asia, Africa, and South America (7, 8).

ST45 was an infrequent type in China. In previous studies, the majority of the ST45-MRSA isolates were found to possess staphylococcal cassette chromosome *mec* type V (SCC*mec*) type V in Hong Kong (9), while there was a clear dominance of SCC*mec* type IV among ST45-MRSA isolates in Hainan (10) and Guangzhou (11), which accounted for 100%. ST45-V was isolated in Hong Kong and Taiwan but had never been found on the mainland of China. Of the 43 ST45-MRSA strains reported in two studies in Hong Kong (9, 12), 39 (90.7%) exhibited t1081, and 4 other isolates exhibited *spa* types (t1768, t1856, and t1861) related to t1081/ST45. In Hainan (10) and Guangzhou (11), ST45-MRSA was associated mainly with t116, with a percentage of 70% and 66.7%, respectively. In recent years, Guangzhou has reported small-scale isolations of MRSA ST45 several times, which attracted our attention (11, 13–15). The mentioned ST45 isolates were all typed as SCC*mec* IV. It turns out that ST45-IV had already become a major clone in MRSA isolates in Guangzhou, China in 2015 to 2018, showing potential to replace ST59 as the predominant MRSA clone in southern China areas (11). Blood samples made up an important proportion of the described MRSA ST45 isolates (11).

In general, reports on Chinese ST45 displayed a deficiency of definite clinical and molecular information, let alone virulence. Given the experience from Europe (4), we envisaged the possibility of the ST45-MRSA isolates being hyper-virulent when causing bloodstream infections. Genomic and phylogenetic analysis of Chinese ST45-MRSA strains is lacking. This study attempts to fill the gap in these areas. We first launched a retrospective investigation to basically understand the prevalence, molecular epidemiology, and genetic characteristics of MRSA ST45 through clinical isolates collected from several Chinese hospitals. Then we focused on ST45-SCC*mec* IV and ST45-SCC*mec* V clinical isolates from Guangzhou and explored their phenotypic characteristics of virulence in bloodstream infection and expression of virulence-related genes.

## RESULTS

### Identification of MRSA ST45.

In this study, we collected a total of 565 MRSA isolates retrospectively collected at seven representative hospitals between from 2014 to 2020. The MRSA isolates covered seven geographical districts scattered across mainland China. Sequence types of MRSA strains were confirmed by multilocus sequence typing (MLST). Overall, 27 MRSA clinical isolates verified to be ST45 were identified from the seven collections, accounting for 4.8% (27/565) (16). The majority of ST45-MRSA isolates were detected in Guangzhou, representing 66.7% (18/27), while other isolates were from different regions, including Jiangxi (3/27), Hubei (3/27), Zhejiang (2/27) and Sichuan (1/27). Most of the ST45 isolates were identified from elderly people and young children, together accounting for 85.2% (23/27). Our isolates comprised mixed specimen types. ST45 accounted for 6.7% (13/195) of blood samples, 4.5% (8/178) of pus/wound exudate samples, and 3.1% (6/192) of sputum samples.

### Molecular characteristics of MRSA ST45 isolates.

SCC*mec* type IV was detected in 85.2% of the isolates (23/27), and the other four ST45 isolates carried SCC*mec* V, representing 14.8% (4/27). The most prevalent *spa* type among all ST45-IV isolates was t116, representing 73.9% (17/23), while three ST45-V isolates belonged to *spa* type t1081, and the last one was undetermined.

All ST45 isolates carried the *hla*, *hlb*, *hlg*, and *hld* genes associated with hemolysin, but all isolates lacked the *lukS/F-PV* genes encoding Panton-Valentine leukocidin (PVL). Compared to the isolates of SCC*mec* type V, those SCC*mec* IV isolates carried more virulence genes. Some virulence genes harbored by ST45-MRSA isolates of the two SCC*mec* types differed significantly. As shown in Table 1, the genes *coa*, *sec*, *sell*, *fnbA*, and *sdrD* were frequently found in type IV strains, with a carried percentage of 100% or 73.9%, but only one SCC*mec* V isolate possessed *sdrD* (accounting for 25%). However, the detection rates of *hysA* and *sdrE* genes in SCC*mec* IV isolates were extremely low. Those SCC*mec* V isolates had high rates of harboring *hysA* (100%) and *sdrE* (75%).

**TABLE 1:** Distribution of antimicrobial resistance genes and virulence genes differentially carried by 27 MRSA ST45 isolates of two SCC*mec* types in China^*a*^

Gene	N (%)			*P* value^*b*^
	Total (*n* = 27)	SCC*mec* IV (*n* = 23)	SCC*mec* V (*n* = 4)	
Resistance genes (class of antimicrobial)				
*tetK* (Tet)	4 (14.8)	0 (0.0)	4 (100.0)	<0.0001
*aac6 aph2* (AGly)	4 (14.8)	0 (0.0)	4 (100.0)	<0.0001
*blaZ* (Bla)	27 (100.0)	23 (100.0)	4 (100.0)	NA
*ermC* (MLS)	17 (63.0)	16 (69.5)	1 (25.0)	0.128
Virulence genes (encoded protein)				
*coa* (staphylocoagulase)	23 (85.2)	23 (100.0)	0 (0.0)	<0.0001
*sec* (staphylococcal enterotoxin C)	17 (63.0)	17 (73.9)	0 (0.0)	<0.05
*sell* (staphylococcal enterotoxin-like L)	17 (63.0)	17 (73.9)	0 (0.0)	<0.05
*fnbA* (fibronectin-binding protein FnbA)	23 (85.2)	23 (100.0)	0 (0.0)	<0.0001
*hysA* (hyaluronate lyase HysA)	4 (14.8)	0 (0.0)	4 (100.0)	<0.0001
*sdrD* (MSCRAMM family adhesin SdrD)	24 (88.9)	23 (100.0)	1 (25.0)	<0.01
*sdrE* (MSCRAMM family adhesin SdrE)	3 (11.1)	0 (0.0)	3 (75.0)	<0.01

a Tet, tetracycline; AGly, aminoglycoside; Bla, β-lactamase; MLS, macrolide-lincosamide-streptomycin.

b *P* value compares SCC*mec* IV and SCC*mec* V isolates for frequency of resistance genes and virulence genes; Fisher’s exact test. NA, not applicable.

In addition, the 27 ST45-MRSA isolates contained the immune evasion cluster (IEC) system. They were positive for IEC genes *scn* (staphylococcal complement inhibitory protein), *chp* (chemotaxis inhibitory protein), and *sak* (staphylokinase) and lacked enterotoxin gene *sea*, presenting the IEC type B (17).

### Antimicrobial resistance profiles of MRSA ST45 isolates.

The antimicrobial susceptibility results are summarized in Table 2. All ST45 isolates were susceptible to ceftaroline, dalfopristin/quinupristin, trimethoprim/sulfamethoxazole, teicoplanin, linezolid, fusidic acid, vancomycin, daptomycin, and dalbavancin. The resistance rate of erythromycin was the highest (18/27, 66.7%). In total, 37.0% (10/27) of isolates were found to be multidrug resistant (MDR) MRSA, with the SCC*mec* V isolates more likely to be MDR (4/4, mainly involving tetracycline, ciprofloxacin, and gentamicin) than those carrying the SCC*mec* IV element (6/23, mainly involving erythromycin, clindamycin, and rifampicin). ST45-MRSA-V appears to be more resistant than the SCC*mec* IV strains.

**TABLE 2:** Percentage of MRSA ST45 isolates resistant to antimicrobial drugs

Antimicrobial drug	Drug-resistant percentage, *N* (%)			*P* value^*a*^
	Total (*n* = 27)	SCC*mec* IV (*n *= 23)	SCC*mec* V (*n* = 4)	
Cefoxitin	27 (100.0)	23 (100.0)	4 (100.0)	NA
Ceftaroline	0 (0.0)	0 (0.0)	0 (0.0)	NA
Ciprofloxacin	5 (18.5)	1 (4.3)	4 (100.0)	<0.001
Clindamycin				
Resistant^*b*^	4 (14.8)	2 (8.6)	2 (50.0)	0.092
D-test positive^*c*^	14 (51.9)	14 (60.9)	0 (0.0)	<0.05
Erythromycin	18 (66.7)	16 (69.6)	2 (50.0)	0.582
Gentamicin	4 (14.8)	0 (0.0)	4 (100.0)	<0.0001
Rifampin	6 (22.2)	6 (26.1)	0 (0.0)	0.5459145
Tetracycline	4 (14.8)	0 (0.0)	4 (100.0)	<0.0001
QDA (dalfopristin/quinupristin)	0 (0.0)	0 (0.0)	0 (0.0)	NA
SXT (trimethoprim/sulfamethoxazole)	0 (0.0)	0 (0.0)	0 (0.0)	NA
Teicoplanin	0 (0.0)	0 (0.0)	0 (0.0)	NA
Linezolid	0 (0.0)	0 (0.0)	0 (0.0)	NA
Fusidic acid	0 (0.0)	0 (0.0)	0 (0.0)	NA
Vancomycin	0 (0.0)	0 (0.0)	0 (0.0)	NA
Daptomycin	0 (0.0)	0 (0.0)	0 (0.0)	NA
Mupirocin	2 (7.4)	0 (0.0)	2 (50.0)	<0.05
Dalbavancin	0 (0.0)	0 (0.0)	0 (0.0)	NA
>3 Non-β-lactam antimicrobial drugs	10 (37.0)	6 (26.1)	4 (100.0)	<0.05

a *P* value compares SCC*mec* IV and SCC*mec* V isolates for resistance to each antimicrobial drug or test; Fisher’s exact test. NA, not applicable.

b This row represents single-agent testing results for clindamycin.

c This row represents D test results for 14 isolates that were erythromycin resistant and clindamycin susceptible.

Interestingly, there were discrepancies as to the resistance profile between ST45-MRSA-IV and MRSA-V. None of the ST45-SCC*mec* IV isolates were susceptible to rifampicin (RIF), exhibiting intermediate resistance or resistance, whereas all ST45-SCC*mec* V isolates were susceptible to RIF. The 23 ST45-SCC*mec* IV isolates were susceptible to tetracycline and gentamicin, while the four ST45-SCC*mec* V isolates were resistant to the two antimicrobials. Only one ST45-SCC*mec* IV isolate was ciprofloxacin resistant (1/23, 4.3%); however, the resistance rate of ST45-SCC*mec* V to ciprofloxacin was 100% (4/4). A total of 14 erythromycin-resistant and clindamycin-susceptible isolates belonged to ST45-SCC*mec* IV and were positive by D test.

As listed in Table 1, all isolates harbored an antimicrobial resistance gene conferring resistance to β-lactam (*blaZ*). The ST45-SCC*mec* V isolates possessed *aac6*/*aph2* and *tetK* genes, which were absent in ST45-SCC*mec* IV isolates. The presence of these genes gives the strains phenotypic resistance to aminoglycosides (*aac6*/*aph2*) and tetracycline (*tetK*).

### Phylogenetic and comparative genomic analysis.

**FIG 1:**
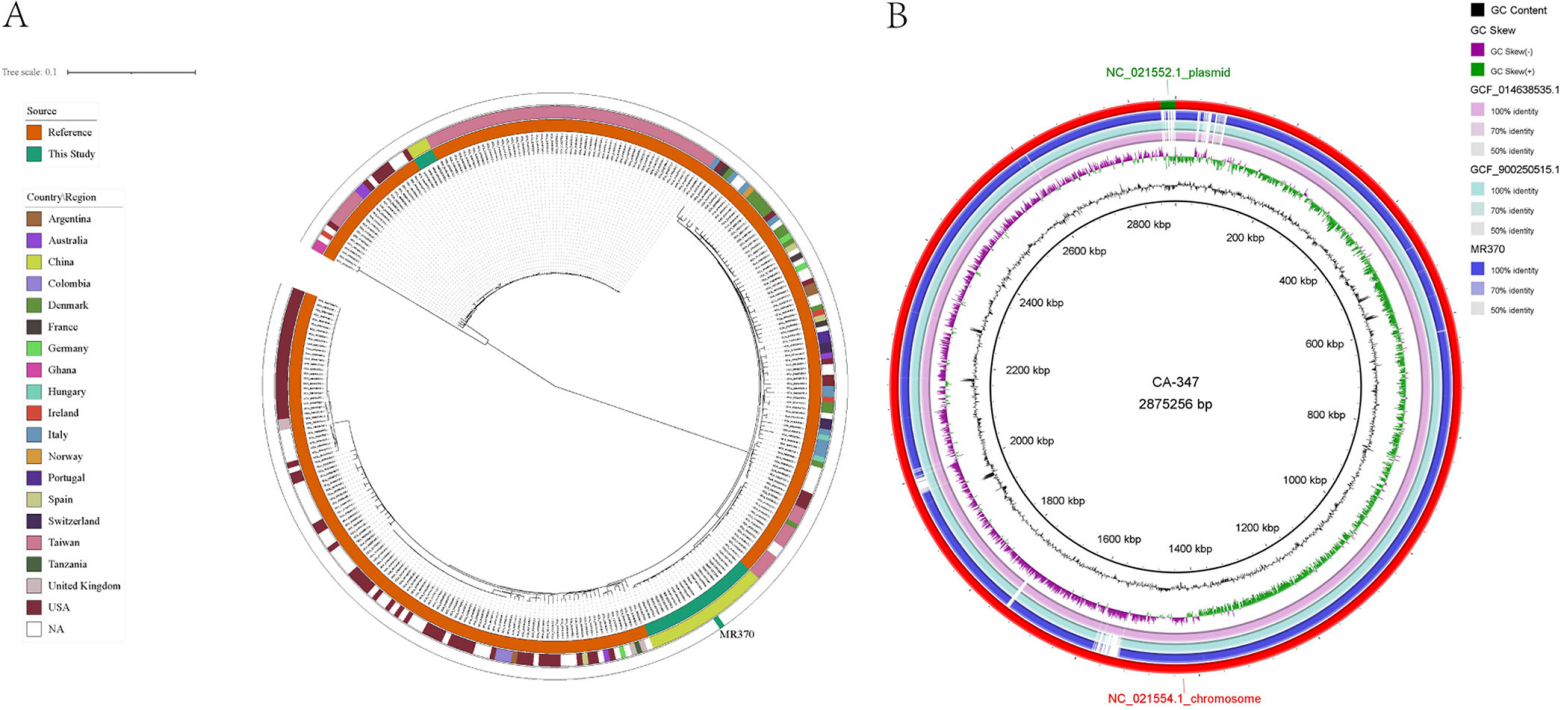
Analysis of the genomes of 27 MRSA ST45 isolates. (A) ParSNP phylogenetic tree of 308 S. aureus ST45 genomes from the RefSeq database and the present study. (B) Comparison of the genomes of MR370 and other MRSA ST45 strains.

The core single nucleotide polymorphism (SNP) phylogenetic analysis showed that all 27 ST45-MRSA isolates formed two definitely separated clusters with 23 SCC*mec* IV strains located in one clade and four SCC*mec* V strains in another (Fig. 1), indicating the breakout of MRSA ST45 clonotypes. The 23 ST45-IV strains differ from each other by 431 (range, 3 to 1623) core SNPs. This SCC*mec* IV clade was close to a clade consisting of 1 Danish, 2 unsourced, and 12 Taiwanese ST45 strains, together in the same evolutionary branch. The 4 strains of SCC*mec* V differ from each other by 724 (range, 45 to 1,119) core SNPs and were most closely related to a collection of ST45 from Taiwan (*n* = 55), followed by those from the United States (*n* = 6) and Australia (*n* = 2). Compared with the recognized MRSA ST45 strain USA600 (GenBank accession no. CP006044), the 23 ST45-IV strains had an average of 2,651 core SNPs (range, 1,963 to 3,586), and the 4 ST45-V had a 15,498 (range, 14,929 to 16,048)-core SNP difference.

When we explored the genetic relationship of our strain, MR370 turned out to be a representative strain which matched the features of most ST45-MRSA isolates and occupied the SCC*mec* IV cluster; two MRSA ST45 strains (from Denmark GCA_900250515.1 and from Taiwan GCA_014638535.1) were selected in accordance with the maximum likelihood tree, and the reference strain CA-347 (a USA600 strain, GenBank accession no. CP006044) was added to construct genomic comparisons. MR370 varied from the Danish strain with a 389-core SNP discrepancy and from the Taiwanese strain by 397 core SNPs. MR370 and CA-347 were relatively far away on the evolutionary tree, with a 1,963-core SNP difference. Besides the SNP variation, comparative genome analysis showed that MR370 had some regions of deletion compared with CA-347. The differential regions included the SCC*mec* type II region and partial phage regions after mobile element analysis (Fig. 1).

### Characteristics of bacterial isolates MR370 and MR387.

The representative MR370 was recovered from the venous blood of a 5-year-old child from Guangzhou, China, in January 2017. We identified MR370 as an ST45-SCC*mec* IV-t116 clinical isolate. MR387 was one of the minority which belonged to ST45-SCC*mec* V-t1081, isolated from the venous blood of a 73-year-old person in Guangzhou, China, in May 2017. MR370 and MR387 lack the *lukS/F-PV* genes. We decided to explore the virulence phenotype of ST45-SCC*mec* IV strain MR370 and SCC*mec* V strain MR387 in bloodstream infections. To establish a comparison, we selected MRSA strains also isolated from blood specimens belonging to prevalent Chinese sequence types CA-MRSA ST59 and HA-MRSA ST5. Furthermore, USA300-LAC, a prototypic S. aureus ST8 strain of the CA-MRSA clone USA300 from the United States, was used for comparison.

### Hemolysin activity determination.

**FIG 2:**
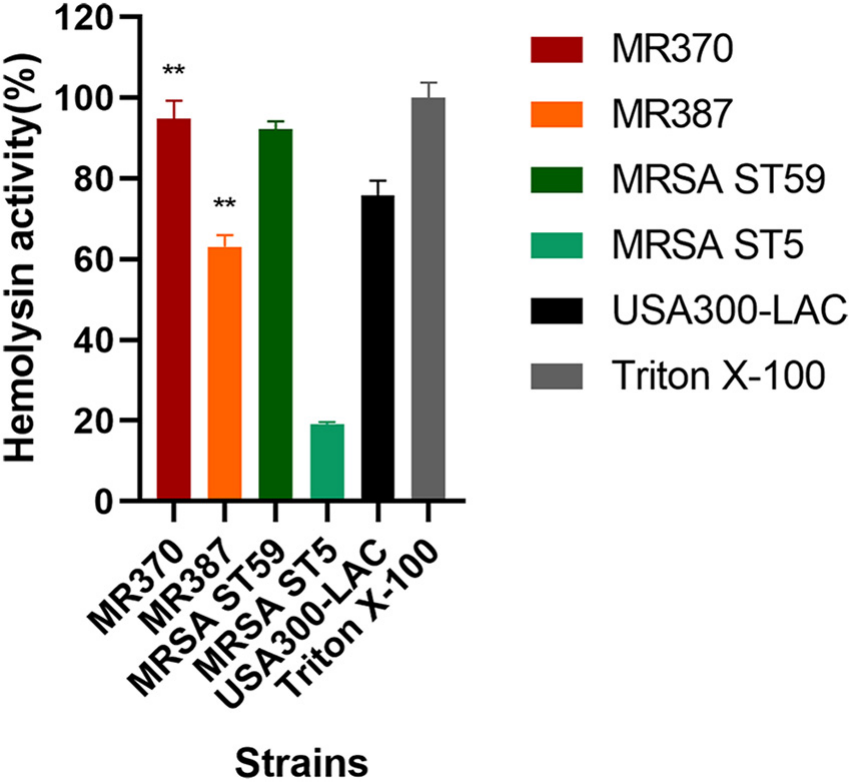
Hemolysis phenotype of methicillin-resistant S. aureus strains. Hemolysin activity was compared by converting A_600_ to a percentage of the positive control (Triton X-100). **, *P *< 0.01 compared with USA300-LAC (unpaired *t* test).

As shown in Fig. 2, MR370 had the highest hemolysis ability among these MRSA strains, which was significantly stronger than that of the ST5 strain (*P < *0.0001) and the USA300-LAC strain (*P < *0.01). The hemolytic abilities of the ST45-MRSA strain MR370 and the ST59-MRSA strain were comparable. Another MRSA ST45 strain, MR387, exhibited less hemolysis, which was weaker than that of USA300-LAC (*P < *0.01) but higher than the ST5 clone strain (*P < *0.0001).

### Whole-blood killing assay.

**FIG 3:**
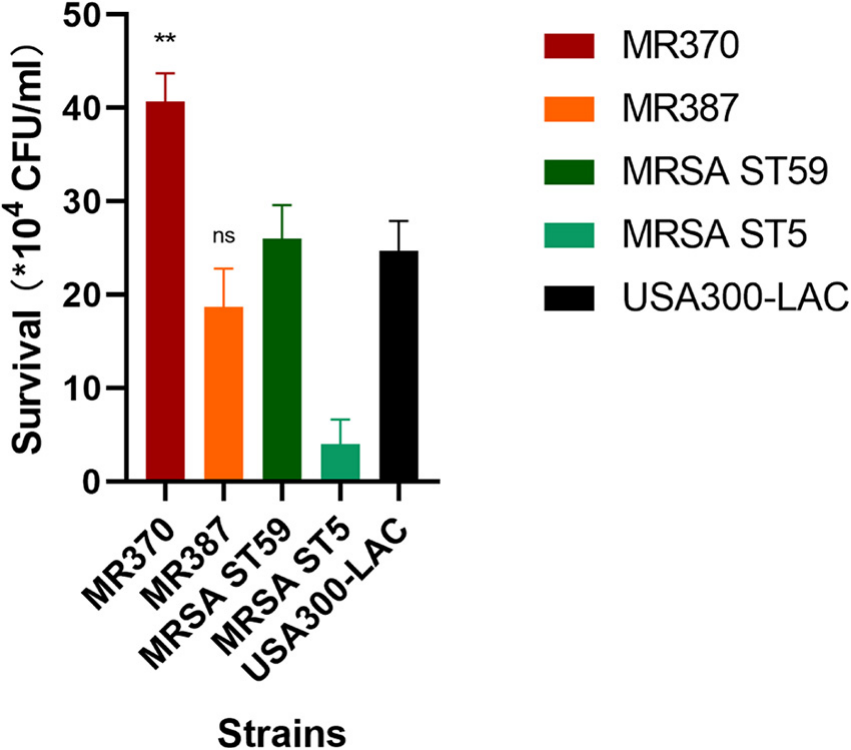
The comparison of bacterial survival in human whole blood between five MRSA strains. **, *P* < 0.01 compared with USA300-LAC (unpaired *t* test); ns, not significant.

The five MRSA strains were then analyzed in a whole-blood killing assay, and the survival ability of MR370 was the strongest (Fig. 3). MR370 showed almost a 10-fold bacterial survival in human blood compared with the MRSA ST5 strain (*P < *0.0001). Compared with the MRSA ST59 strain and the USA300-LAC strain, MR370 exhibited significantly higher survival (*P < *0.01). MR387 exhibited comparable survival ability with USA300-LAC (ns). The survival ability of MR387 was slightly lower than that of the MRSA ST59 strain (ns) but much higher than that of the ST5 clone strain (*P < *0.01).

### Galleria mellonella infection model.

**FIG 4:**
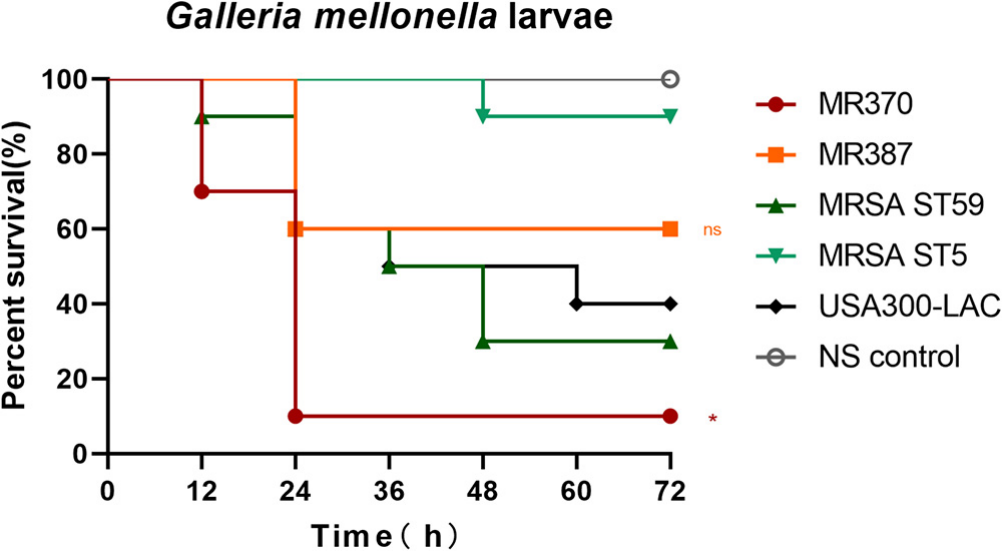
Virulence in the Galleria mellonella wax moth larva infection model. The survival rates of infected larvae are shown as Kaplan-Meier survival curves. *, *P < *0.05 compared with USA300-LAC (log-rank test); ns, not significant.

We compared survival rates of Galleria mellonella infected with the five MRSA strains to initially analyze the virulence *in vivo* in bloodstream infections (Fig. 4). As expected, MR370 caused the lowest larval survival rate, showing statistical significance in comparison with that infected with USA300-LAC (*P < *0.05). Survival of G. mellonella larvae dropped to 10% only 1 day after infection with MR370. In contrast, the survival rate of larvae infected with the MRSA ST5 strain remained high throughout the experiment (*P < *0.001 compared with MR370). Although the MRSA ST59 strain led to higher survival of G. mellonella larvae than MR370, the difference was not significant, indicating similar virulence of the two MRSA strains in this model. Another MRSA ST45 strain, MR387, led to moderate survival (60% after 24 h) among all strains. The survival rates of larvae infected with MR387 and USA300-LAC were comparable (ns), indicating their similar virulence in this model.

### Mouse bacteremia model.

**FIG 5:**
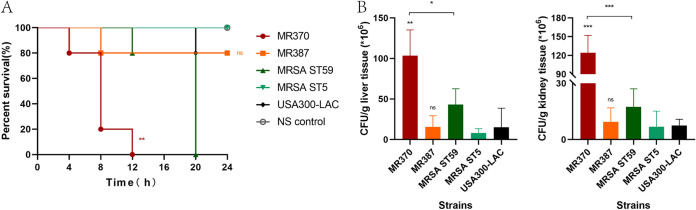
Infections caused by five MRSA strains in the mouse bacteremia model. Five mice were used per strain. (A) Survival analysis. Comparison of survival curves was established by a log-rank (Mantel-Cox) test. **, *P < *0.01 compared with USA300-LAC; ns, not significant. (B) CFU counts in kidney and liver was determined. *, *P < *0.05; **, *P < *0.01; ***, *P < *0.001 (unpaired *t* test); ns, not significant.

Subsequent to the Galleria mellonella infection model, the pathogenicity of MR370 and MR387 in bloodstream infection *in vivo* was further evaluated in a mouse bacteremia model. As shown in Fig. 5A, the mice infected with MR370 all died within 12 h, while the MRSA ST59 strain caused a 100% mortality in mice at the 20-h point. However, the percentage of surviving mice was consistently 100% following infection with the MRSA ST5 strain. USA300-LAC resulted in the death of one mouse at the 20-h point. Compared with the other three MRSA strains, infection with MR370 produced significantly greater mortality (*P < *0.01). The other MRSA ST45 strain, MR387, and the USA300-LAC strain resulted in similar survival in mice (ns). As shown in Fig. 5B, CFU counts in the kidneys and livers were the highest in the mice infected with the high-virulence ST45-SCC*mec* IV strain MR370, while the MRSA ST5 strain caused the lowest bacterial load of tissues in mice (kidney, *P < *0.01; liver, *P < *0.01). The MR370 group reached a staggering order of magnitude of 10^8^. The mice infected with MR370 were found to have higher CFU counts in the kidneys and livers than those infected with the MRSA ST59 strain (kidney, not significant; liver, *P < *0.05) and USA300-LAC (kidney, *P < *0.05; liver, *P < *0.01). The bacterial burden of tissues in the MRSA ST59 group was also heavy, with the number of CFU lower than only the MR370 group, suggesting its strong ability in inducing bacteremia. As for the ST45-SCC*mec* V strain, MR387 and USA300-LAC resulted in comparable CFU counts in the kidneys and livers of infected mice (ns).

In short, MR370 showed strong virulence in all phenotypic experiments, whereas MR387 exhibited similar levels to USA300-LAC. These findings demonstrated the high virulence potential of the two ST45-MRSA isolates to cause bloodstream infections.

### Real-time fluorescence quantitative PCR.

**FIG 6:**
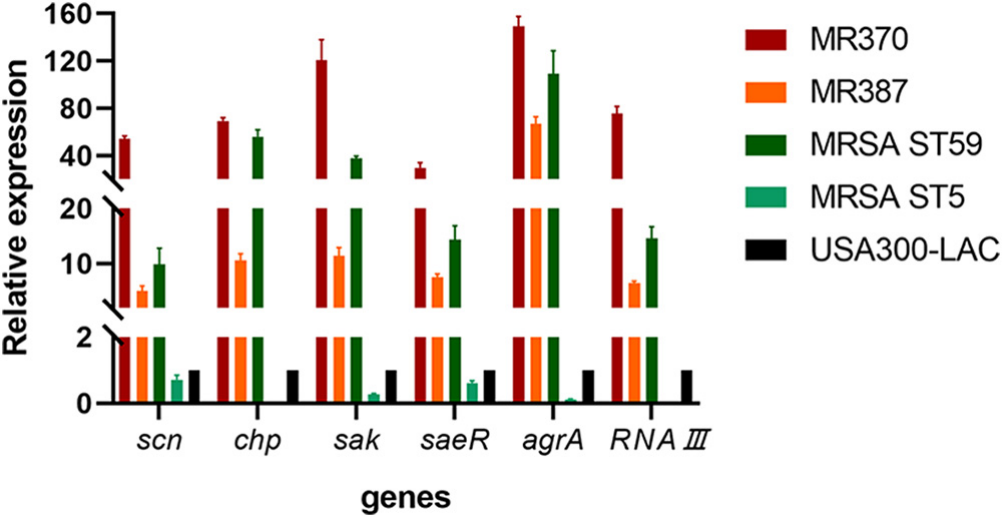
Relative expressions of *scn*, *chp*, *sak*, *saeR*, *agrA*, and *RNAIII* in four MRSA isolates with USA300 as the reference strain. The *chp* gene is absent in the MRSA ST5 strain.

It is necessary to understand the pathogenicity of the two ST45-MRSA isolates. Reverse transcriptase quantitative PCR (RT-qPCR) was carried out to explore the expression of some virulence genes (Fig. 6). The IEC genes MR370 and MR387 were validated first, and the same trend was observed. The expression levels of *scn*, *chp*, and *sak* in MR370 were much higher than those in other strains. Compared to the second most highly expressed MRSA ST59 strain, MR370 still had a significant difference with *scn* (*P < *0.0001), *chp* (*P < *0.05), and *sak* (*P < *0.01). Compared with USA300-LAC, MR370 showed a more significant difference with *scn* (*P < *0.0001), *chp* (*P < *0.0001), and *sak* (*P < *0.001). The expression of IEC genes in MR387 was lower than that in MR370. However, the *scn* expression of MR387 was higher than that of USA300-LAC (*P < *0.01) and similar to that of the MRSA ST59 strain. The *chp* and *sak* expressions of MR387 were much higher than those of USA300-LAC (*P < *0.001). The expression of regulatory genes, *saeR*, *agrA* and *RNAIII*, was also examined. We found that the levels of *saeR*, *agrA*, and *RNAIII* expression in MR370 and MR387 were significantly higher than that in USA300-LAC, with statistical differences of at least *P < *0.001. MR370, in particular, was the strain with the highest expression and showed statistical differences with the MRSA ST59 strain (*saeR*, *P < *0.01; *agrA*, *P < *0.05; *RNAIII*, *P < *0.0001). In total, the relative expression of these virulence-associated genes in MR370 showed a range of 29.71-fold to 149.07-fold, while MR387 exhibited a range of 5.06-fold to 66.68-fold compared to USA300-LAC. The results were indicative of these strains excellent virulence potential and were consistent with the above-described phenotype.

## DISCUSSION

ST45-MRSA-II (USA600) is the most frequently reported ST45 clone in the literature, but it hardly appears outside North America (4). This clone exhibited high survival rates in blood and was thereby closely associated with bacteremia and increased patient mortality (18–20). ST45-MRSA-IV (well-known as Berlin-IV clone), initially observed in Berlin in 1993 (21) and then predominant in Europe (22), was clonally related to USA600 (19). ST45-SCC*mec* IV was able to cause high mortality in bloodstream infections and had a large global spread capacity (23). In Australia, ST45-MRSA-IV and MRSA-V were two of the major indigenous lineages (6). A previous study lends support to the notion that virulence could contribute to pathogen transmission of MRSA strains, thereby providing an explanation for its epidemic (24). Nevertheless, the virulence potential of bloodstream infection of Chinese MRSA ST45 has not been experimentally proven yet. Recently, the emergence of a collection of ST45-SCC*mec* IV and -SCC*mec* V clinical isolates in China caught our attention.

In order to predict whether the ST45-MRSA clone will spread across the country, we analyzed past reports and conducted a new epidemiological investigation throughout China. In retrospect, MRSA ST45 has successfully established itself in the general population in Hong Kong (9, 12, 25, 26) and Taiwan (21, 27) and even accounted for an important proportion of clinical MRSA isolates. Recently, the detection of an MRSA ST45 strain has occurred in southern provinces of China (10, 11, 13–15, 28). Between 2015 and 2018, ST45 constituted 18.8% (12/64, rank third) among clinical MRSA isolates collected from four centers in Guangzhou (11), showing an increase compared to 1.7% in MRSA isolates from Chinese children in an earlier study (13). In 2018, ST45 accounted for 33 (33.5%) of 93 MRSA isolates collected from fecal samples of pediatric patients in three centers in Guangzhou (14). In another prospective, cross-sectional study involving nasal swabs of school-age children in Guangzhou in 2018, ST45 was the number one sequence type among MRSA isolates (34/72) (15). The above-described studies pointed to a conclusion that MRSA ST45 gradually expanded and became widespread in Guangzhou. Besides Guangzhou, MRSA ST45 was only detected in Hainan (10) and Liuzhou (28). In Hainan, ST45 accounted for 26.3% (20/76) of MRSA isolates derived from diverse clinical specimens from three hospitals in 2013 to 2014 and 2018 to 2019 (10). In Liuzhou, two MRSA ST45 isolates were identified from spontaneous ear pus drainage from 2013 to 2015 (28). No other regions have reported the isolation of MRSA ST45 at present. We found that most of these studies mentioned ST45 only when screening and identifying clinical MRSA isolates from a variety of specimens, with some studies providing incomplete molecular information.

The identified MRSA ST45 strains in the present study were mainly concentrated in Guangzhou, which is consistent with the previous epidemic characteristics on the mainland. Notably, ST45-MRSA was also isolated in other regions where ST45 had never been reported before, possibly implicating its importation into the whole of China. Despite the dominance of ST45-SCC*mec* IV, we identified the emergence of SCC*mec* V type with the discovery of four isolates recovered from adult patients in Guangzhou in 2017 and 2018. Phylogenetically, these sporadic SCC*mec* V strains were aggregated in a clade. Thus, we inferred that our strains were closely related to Guangzhou’s. However, the genomes were not available in the database. Phylogenetic analysis indicated that Taiwan was the most likely source of our MRSA ST45 strains. Noticing the molecular type of these isolates, we found SCC*mec* IV-t116 to be the main type. It was concentrated in Guangzhou, and MR370 happened to be one of these epidemic clone strains. Likewise, of the total MRSA ST45 strains detected prior to our study, a substantial proportion belonged to SCC*mec* IV-t116. But ST45-IV-t116 was previously limited to Hainan and Guangzhou. In our survey, the data showed the first identification of ST45-SCC*mec* IV-t116 in Jiangxi, Hubei, and Sichuan provinces. In spite of the small quantity, this phenomenon indicated its inroads northward in China, which could be called an epidemiological breakthrough of ST45- IV-t116. As for the SCC*mec* V strains, *spa* t1081 type was the only classification except for one unknown isolate. This is the first example of ST45-SCC*mec* V-t1081 appearing in mainland China. Guangzhou, as the only source of these new-found SCC*mec* V strains, may be the start of its epidemic in mainland China in the future. Moreover, SCC*mec* V-t1081 was the main type of MRSA ST45 in Hong Kong, and its emergence can be traced back to 2005 (9). It could be speculated that our ST45/SCC*mec* V isolates were connected to Hong Kong. Furthermore, the SCC*mec* element variations and diverse *spa* types in the ST45 clone probably reflect active and extensive genetic recombination. Overall, our data led us to the conclusion that MRSA ST45 was spreading and expanding throughout China, deserving special attention. Therefore, active surveillance should be enacted for the further epidemic spread of MRSA ST45 in China.

It is worth emphasizing that ST45-SCC*mec* IV and -SCC*mec* V isolates separately clustered together, forming two independent evolutionary branches. ST45 strains of SCC*mec* IV were mostly from Guangzhou (14/23, 60.9%), and were also found piecemeal in other areas in China (Jiangxi, Hubei, Zhejiang, and Sichuan). This indicated that the ST45-IV clonotype was spreading in different regions of China. ST45 strains of SCC*mec* V were all isolated from Guangzhou, suggesting a small outbreak of the ST45-V clonotype. Moreover, this may be a strong hint of the dissemination in China of these two clonotypes. Actually, MRSA strains of ST45 with SCC*mec* type II were recognized to exist in Hong Kong around the year 2000, and no SCC*mec* IV or V isolates were collected (29), supporting the view that the appearance of the SCC*mec* II population predated SCC*mec* IV or V among the ST45 strains. However, the ST45/SCC*mec* II clone has not been introduced into the mainland to date. Currently, its phylogenetic regularity is unknown. Given its host diversity and transmission risk (5), it is hard to rule out the possibility of it entering the mainland later.

The high possibility of MRSA ST45 achieving epidemiological success in China like the prominent clone ST59 prompted us to study its virulence. The ST45-SCC*mec* IV strain MR370 demonstrated remarkable virulence potential in hemolysin activity, whole-blood survival, and different animal models, exceeding the other four strains. As a widespread CA-MRSA lineage in Asia, the ST59 clone had pronounced virulence (30). Our results suggested that the MRSA ST59 strain was second only to MR370 in virulence causing bloodstream infection. ST5 was one of the major international EMRSA STs (31). Belonging to HA-MRSA, the ST5 clone presented significantly weaker virulence than others, as expected (30). USA300, the epidemic CA-MRSA clone in America, was among the most virulent strains (24, 32). In a recent study in Taiwan (33), 92 USA300 isolates were identified among 232 MRSA bloodstream isolates, with a proportion of 39.7%. Compared with the USA300-LAC strain, we found MR370 to be much more virulent. Although the other MRSA ST45 strain, MR387 (SCC*mec* V), did not perform as well as MR370 (SCC*mec* IV), it had similar behavior to USA300-LAC in all the assays, indicating its strong virulence. Taken together, there was consistent evidence that these two MRSA ST45 isolates had high virulence capacity in terms of bloodstream infections. It is not known what genetic factors underlie the hyper-virulence. We intended to trace the cause of the virulence phenotypes at mRNA level. All 27 MRSA ST45 isolates were shown to contain IEC type B (*scn*, *chp*, and *sak*), so we hypothesized that the IEC system may play a role in immune defenses and achieve the strains’ virulence. The results showed that the relative expression of *scn*, *chp*, and *sak* in MR370 and MR387 was indeed much higher than that of USA300-LAC, which contained IEC type B too. The expression of virulence factors is controlled by a network of regulatory systems, such as *agr* and *saeRS*. The transcript *RNAIII* is encoded by *agr* locus, and *agrA* is an essential transcription factor for *RNAIII* (34). The *saeR*, *agrA*, and *RNAIII* expression of MR370 and MR387 was significantly higher than that of USA300-LAC. It is tempting to speculate that the high-level virulence of our MRSA ST45 strains may result from these virulence gene regulators in a coordinated fashion.

The limitation is that our study did not address the detailed virulence mechanism. This is one issue that deserves follow-up in future studies. The specific virulence regulatory mechanism warrants further investigation.

In summary, ST45-IV and ST45-V clonotypes occurred and spread in mainland China. The profound ability for widespread dissemination of MRSA ST45 was a wake-up call for us. Moreover, we reported for the first time the identification and analysis of ST45-SCC*mec* V on the mainland as well as virulence of MRSA ST45 in China. Our findings revealed the strong ability of ST45- SCC*mec* IV isolate MR370 and SCC*mec* V isolate MR387 to induce bloodstream infection. Carefully monitoring the emergence of MRSA ST45 is crucial for investigation and prevention strategies. Additional surveillance of environmental samples will be necessary. Our study provides novel insights for preventing the occurrence and spread of these strains.

## MATERIALS AND METHODS

### Ethics statement.

This study was approved by the Ethics Committee of Shanghai Pulmonary Hospital, School of Medicine, Tongji University, Shanghai, China. Individual patients or their legal guardians provided informed consent. Animal assays were approved by the Institutional Animal Care and Use Committee of Shanghai Pulmonary Hospital, School of Medicine, Tongji University, Shanghai (project number K19-028Y).

### Collection of bacterial isolates.

The clinical methicillin-resistant S. aureus isolates were collected from seven regions located in different provinces and municipalities of China: Shanghai, Zhejiang, Hubei, Jiangxi, Sichuan, Guangdong, and Inner Mongolia. The isolates were recovered from separate locations of patients: blood (34.5%, 195/565), pus/wound exudate (31.5%, 178/565), and sputum (34.0%, 192/565). All MRSA isolates were classified into 28 STs, with ST59 (27.1%, 153/565) and ST5 (11.0%, 62/565) most frequently identified (16). The bacterial isolates were cultured in routine microbiology laboratories. Their species were reidentified by matrix-assisted laser desorption ionization–time of flight mass spectrometry (MALDI-TOF MS; Bruker Daltonics GmbH, Bremen, Germany). All strains were confirmed as MRSA through testing cefoxitin susceptibility according to the recommendations of the Clinical and Laboratory Standards Institute (35) and carried the *mecA* gene.

### WGS and analysis of MRSA ST45 isolates.

ST45 was identified by multilocus sequence typing (MLST) from all MRSA isolates. Whole-genome sequencing (WGS) of MRSA ST45 isolates was performed by United Medical Technology Co., Ltd., Shenzhen, China, on the Illumina HiSeq platform. The total DNA of isolates was extracted using a Ezup Column bacterial genomic DNA purification kit (Sangon Biotech, Shanghai, China). The raw data were filtered using Trimmomatic v0.39 (36), followed by assembly using SPAdes v3.15.2 (37). Meanwhile, we characterized all MRSA ST45 isolates by staphylococcal chromosome cassette *mec* (SCC*mec*) types and *spa* types, determined using SCCmecFinder (38) and SpaFinder (39), respectively. The detection of antimicrobial resistance determinants was conducted using AMRFinderPlus v3.10.5 (40). Virulence genes were mined using BLAST against the VFDB database (mgc.ac.cn/VFs/).

### Phylogenetic and comparative genomic analysis.

To analyze the phylogenetic relationship of our 27 ST45-MRSA genomes, S. aureus genome assemblies (*n* = 13,577) were downloaded from the NCBI RefSeq database, and the ST45 assemblies (*n* = 308) were then extracted after determination of the MLST with mlst v2.19.0 (github.com/tseemann/mlst). The core SNP phylogenetic tree was generated using ParSNP from Harvest v1.12 (41), followed by visualization and annotation using iTOL v6 (42). Core SNP analysis was conducted using the S. aureus USA600 strain CA-347 genome (accession no. CP006044) as the reference. Comparative genomic analysis was established, and BLAST Ring Image Generator (BRIG) was used to compare the reference strain CA-347 genome with other representative genomes to further generate circular maps.

### Antimicrobial susceptibility testing.

According to the protocols provided by the Clinical and Laboratory Standards Institute (35), the antimicrobial susceptibility of ST45-MRSA isolates to 18 antimicrobial agents was determined. Ceftaroline, erythromycin, clindamycin, tetracycline, ciprofloxacin, dalfopristin/quinupristin, and cefoxitin were tested by disk diffusion in Mueller-Hinton agar plates (Oxoid, UK). The broth microdilution method was used to determine the MICs of trimethoprim-sulfamethoxazole, gentamicin, daptomycin, mupirocin, rifampicin, teicoplanin, linezolid, fusidic acid, vancomycin, dalbavancin, and cefoxitin. The D test was performed to determine inducible clindamycin resistance. S. aureus ATCC 29213 and ATCC 25923 strains were used for quality control. Isolates resistant to three or more non-β-lactam antimicrobial drugs were defined as multidrug resistant (MDR) in this study (43).

### Hemolysin activity determination.

The S. aureus strains were incubated in Trypticase soy broth (TSB) at 37°C overnight in a shaker. Supernatants were collected from bacterial cultures grown for 24 h with optical density values normalized at 600 nm. Hemolytic activities were determined by incubating supernatant of selected strains with 3% sterile defibrillation rabbit blood in phosphate-buffered saline (PBS) for 1 h at 37°C. Ultimately, the red blood cells deposited were discarded after centrifugation, and hemolysis was semiquantified by measuring the optical density of the supernatant at 600 nm. Triton X-100 was used as a positive control. The assay was performed in triplicate.

### Whole-blood killing assay.

The S. aureus strains were grown in TSB to the postexponential phase. Then approximately 10^6^ CFU of bacteria were suspended to fresh heparinized human whole blood for incubation at 37°C for 6 h with constant rotation. Serially diluted samples were plated onto nutrient agar plates to enumerate the surviving bacteria. The assay was repeated three times.

### Galleria mellonella infection model.

Mono-clone of S. aureus was suspended into TSB and cultured in a shaking table for 12 h at 37°C and 220 rpm. Then strains were reactivated to the logarithmic phase. Bacterial deposits were collected after centrifugation and complete discarding of the supernatant and were adjusted with normal saline to 1.5 × 10^8^ CFU/mL. Microsyringes were used to inject different S. aureus strains in a volume of 10 μL into Galleria mellonella wax moth larvae. A group of 10 healthy larvae were chosen for each strain. Treated larvae were placed in an air incubator at 37°C and observed at regular intervals for 72 h. Mortality rates among different strains were compared to measure their virulence. Each test was performed independently in triplicate.

### Mouse bacteremia model.

Six-week-old, female BALB/C mice under a stable raising condition were used for the experiment. Overnight cultures (grown in TSB) of selected strains were subcultured (1:100) in fresh TSB for growing to the logarithmic phase. Bacterial cells were washed twice and resuspended in sterile normal saline, followed by intraperitoneal injection with 100 μL saline containing 1 × 10^9^ CFU per mouse. After inoculation, mouse health and disease advancement were monitored continuously for survival analysis, and mice were dissected immediately if any of the mice died. All surviving mice were euthanized at 24 h. Kidney and liver were excised and homogenized for CFU determination. S. aureus CFU was assessed by plating serially diluted homogenized tissue on nutrient agar plates.

### Real-time fluorescence quantitative PCR.

The S. aureus strains were cultured overnight at 37°C with shaking. Based on the kit instructions, the total RNA was extracted using a Spin Column bacterial total RNA purification kit (Sangon Biotech, Shanghai, China). Then the cDNA was synthesized from purified total RNA using a PrimeScript RT reagent kit (TaKaRa, Japan). Subsequently, real-time PCRs were performed in a 20-μL system including cDNA, primers, and TB Green premix Ex *Taq* II (TaKaRa, Japan). The relative expression level of the target gene was calculated using the formula 2^−ΔΔ^*^CT^*, and *gyrb* was the internal reference gene and USA300-LAC was the reference strain. Table 3 lists the primer pairs used in this study. Each reaction was performed in triplicate.

**TABLE 3:** Primer pairs used in RT-PCRs

Primer	Primer sequence (5′ → 3′)
*gyrb*-RT-F	ACATTACAGCAGCGTATTAG
*gyrb*-RT-R	CTCATAGTGATAGGAGTCTTCT
*scn*-RT-F	CTATACTTGCGGGAACTT
*scn*-RT-R	TTGATATTCATTCGATGTTGG
*chp*-RT-F	TAACGGCAGGAATCAGTA
*chp*-RT-R	GTTGTAGGAAGACCACTATT
*sak*-RT-F	ACCTGGGACTACACTTACA
*sak*-RT-R	CGCTTGGATCTAATTCAACTAC
*saeR*-RT-F	GTCGTAACCATTAACTTCTG
*saeR*-RT-R	ATCGTGGATGATGAACAA
*agrA*-RT-F	TCCAGCAGAATTAAGAACTCG
*agrA*-RT-R	ATATCATCATATTGAACATACACT
*RNAIII*-RT-F	GCACTGAGTCCAAGGAAACTAAC
*RNAIII*-RT-R	AAGCCATCCCAACTTAATAACC

### Statistical analysis.

GraphPad Prism (v8.0, La Jolla, CA, United States) was used to analyze the data. Unpaired two-tailed *t* tests and the Fisher exact test were performed to evaluate statistical significance. The log-rank test was used to estimate the Kaplan-Meier survival curves. A *P* value of <0.05 was considered statistically significant. Error bars in the graphs indicate the standard deviation (mean ± SD).
